# Environmental and molecular approach to dye industry waste degradation by the ascomycete fungus *Nectriella pironii*

**DOI:** 10.1038/s41598-021-03446-x

**Published:** 2021-12-13

**Authors:** Aleksandra Góralczyk-Bińkowska, Andrzej Długoński, Przemysław Bernat, Jerzy Długoński, Anna Jasińska

**Affiliations:** 1 Department of Industrial Microbiology and Biotechnology, Faculty of Biology and Environmental Protection, University of Łódź, 12/16 Banacha Street, 90-237 Łódź, Poland; 2 Institute of Biological Sciences, Faculty of Biology and Environmental Sciences, Cardinal Stefan Wyszyński University in Warsaw, 1/3 Wóycickiego Street, 01-938 Warsaw, Poland

**Keywords:** Biotechnology, Microbiology, Environmental sciences

## Abstract

Textile industry effluents and landfill leachate contain chemicals such as dyes, heavy metals and aromatic amines characterized by their mutagenicity, cytotoxicity and carcinogenicity. The aim of the present study was investigation of the ascomycete fungus *N. pironii* isolated from urban postindustrial textile green space for its ability to grow and retain metabolic activity in the presence of the dye industry waste. Research focused mainly on dyes, heavy metals and aromatic amines, which had been detected in landfill leachate via HPLC–MS/MS analysis. Presence of all tested compounds as well as leachate in the growth medium clearly favored the growth of fungal biomass. Only slight growth limitation was observed in the presence of 50 mg L^-1^ *o*-tolidine. The fungus eliminated *o*-tolidine as well as dyes at all tested concentrations. The presence of metals slightly influenced the decolorization of the azo dyes; however, it was still similar to 90%. During fungal growth, *o*-tolidine was hydroxylated and/or converted to toluidine and its derivatives. Laccase and cytochrome P450 involvement in this process has been revealed. The results presented in the paper provide a valuable background for the development of a fungus-based system for the elimination of toxic pollutants generated by the textile industry.

## Introduction

**Figure 1:**
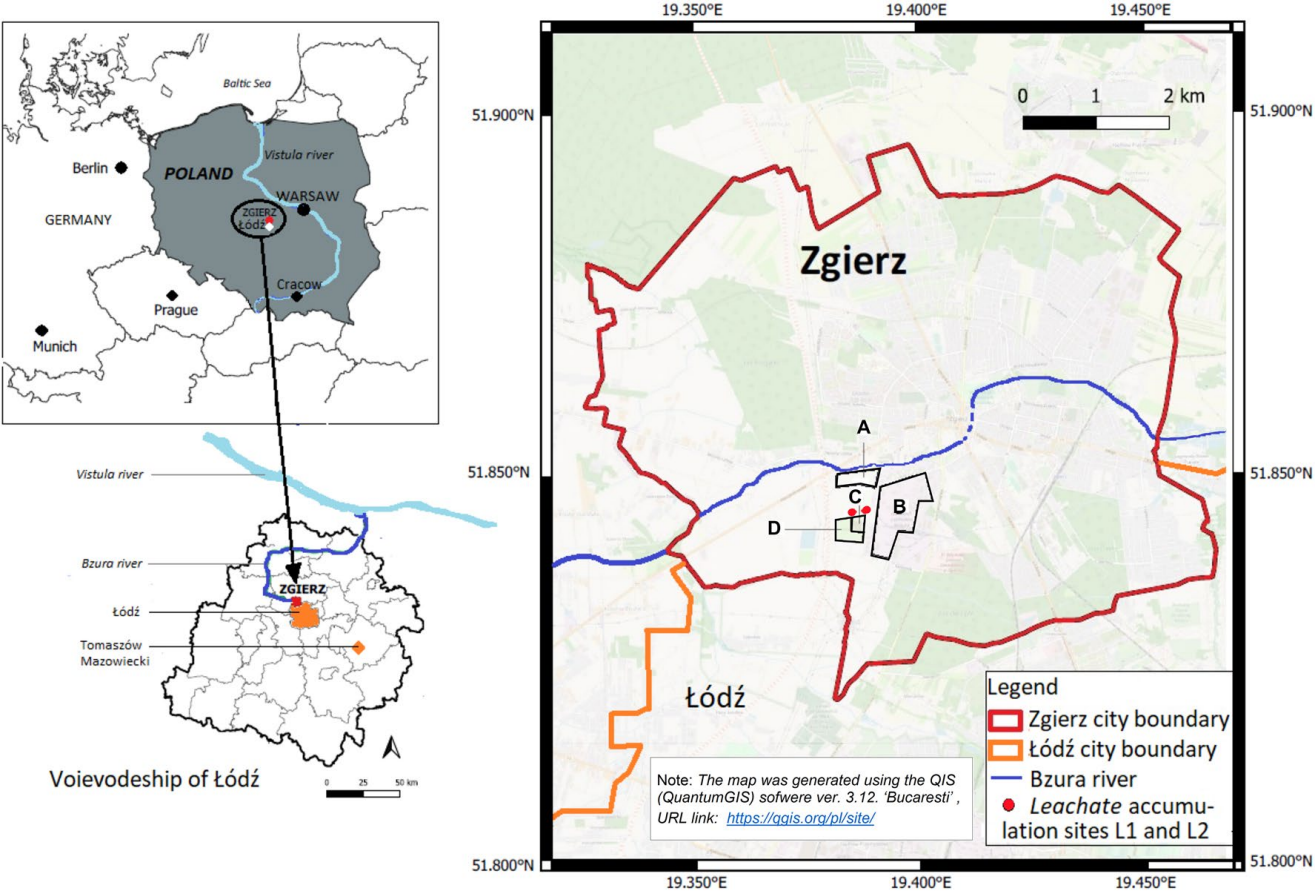
Location of the study region: the municipal and industrial wastewater treatment plant (**A**), the area of the former "Boruta" Dye Industry Plant (**B**), the closed landfill for hazardous waste of the former "Boruta" Dye Industry Plant (**C**), the closed energy ash and gypsum landfill (**D**) (author's own elaboration based on OpenStreetMap data and OSM Standard QuantumGIS).

Civilization development, extensive urbanization and progressive industrialization have contributed to a significant increase in industrial waste in recent years, including toxic pollutants of the dye industry. In 2019, the world dye and pigment market was valued at 33.2 billion USD^1^. The textile industry and related synthetic dye production are also notable sectors of the Polish economy and are hallmarks of the Łódź region in Poland^2–4^. However, it is estimated that approximately 10% of the 70 million tons of synthetic dyes produced annually worldwide are discharged into the environment in the form of process wastewater^5–7^. Industry waste contains a number of deleterious substances and is a key problem both for the environment and for human life and health in numerous countries, including Poland^8–11^. This applies not only to the current production but also to the industrial wastes created in the past. The threat to the environment arises especially there, where larger amounts of waste are improperly accumulated and stored, e.g., in Poland, postproduction waste landfills of the former “Boruta” Dye Industry Plant located in Zgierz near Łódź and the Textile Industry Factory “Wistom” in Tomaszów Mazowiecki (Fig. 1), which are on a list of “Waste collection places that pose a threat to human health and life”^12^.

In the case of landfills for the textile industry, leachate frequently contains synthetic azo dyes, which are commonly used due to the widest scale of colours^7,13,14^ and their intermediates, e.g., potentially carcinogenic aromatic amines, which are formed by anaerobic reduction of these compounds underground. Aromatic amines are highly soluble in water and thus can easily penetrate through the soil and enter the water cycle. Next, the amino group of aromatic amines can be transformed to the reactive intermediate hydroxylamine, which can cause damage to proteins and DNA and thus has been categorized by the International Agency for Research on Cancer as a potential carcinogen^15^. Most important carcinogenic aromatic amines are benzidine and its derivatives, e.g., *o*-tolidine, 3,3’-dichlorobenzidine and 3,3’-dimethoxybenzidine, commonly used for azo dye production. A relationship has been shown between long-term exposure to these amines and the development of bladder cancer^16^. The European Union has banned the usage of azo dyes that release one or more of 22 aromatic amines classified as potential carcinogens in textile materials above a threshold concentration of 30 mg kg^-1^^17^.

Numerous physical and chemical methods as well as their combinations have been developed for deleterious components of textile wastewater elimination. However, these methods are often combined with high operation costs and low efficiency, which make them economically unsuitable, especially for small enterprises^18^. Additionally, during some chemical methods, e.g., oxidation or ozonation, hazardous intermediates are formed^19^. Methods based on biological degradation are more environmentally friendly and less expensive and can be an encouraging alternative for toxic compound elimination in polluted areas^18,20–22^. However, dyeing processes involve not only a variety of colorants but also different alkalis, organic and inorganic salts, acids and heavy metals; hence, it is essential to seek microorganisms that not only decolorize dyes but also retain their properties in conditions unfavorable for the metabolic activity of most microorganisms.

Thus, the proper selection of microbial strains for hazardous waste elimination seems to be one of the most important factors influencing the biodegradation efficiency because only microorganisms adapted to the conditions specific for waste from the dye industry will be fully active in this habitat.

With this in mind, the ascomycete fungus *N. pironii*, previously isolated from urban postindustrial textile green space, was applied in the elimination of dyes and aromatic amines used for their production^23^. The work focused on assessing the suitability of this fungus for the utilization of leachate from a waste landfill of the former “Boruta” Dye Industry Plant in Zgierz (Poland). The biodegradation of *o*-tolidine and azo dyes detected in the landfill leachate has also been demonstrated. Dye decolorization in the presence of heavy metals has been investigated. Preliminary identification of the mechanisms responsible for fungal degradation was carried out. Laccase and cytochrome P450 (CYP) involvement has been revealed, which may allow the design of efficient systems for the elimination of these deleterious contaminants from textile postindustrial areas.

## Results and discussion

### Characteristics of the landfill of the former “Boruta” Dye Industry Plant and the landfill leachate

**Figure 2:**
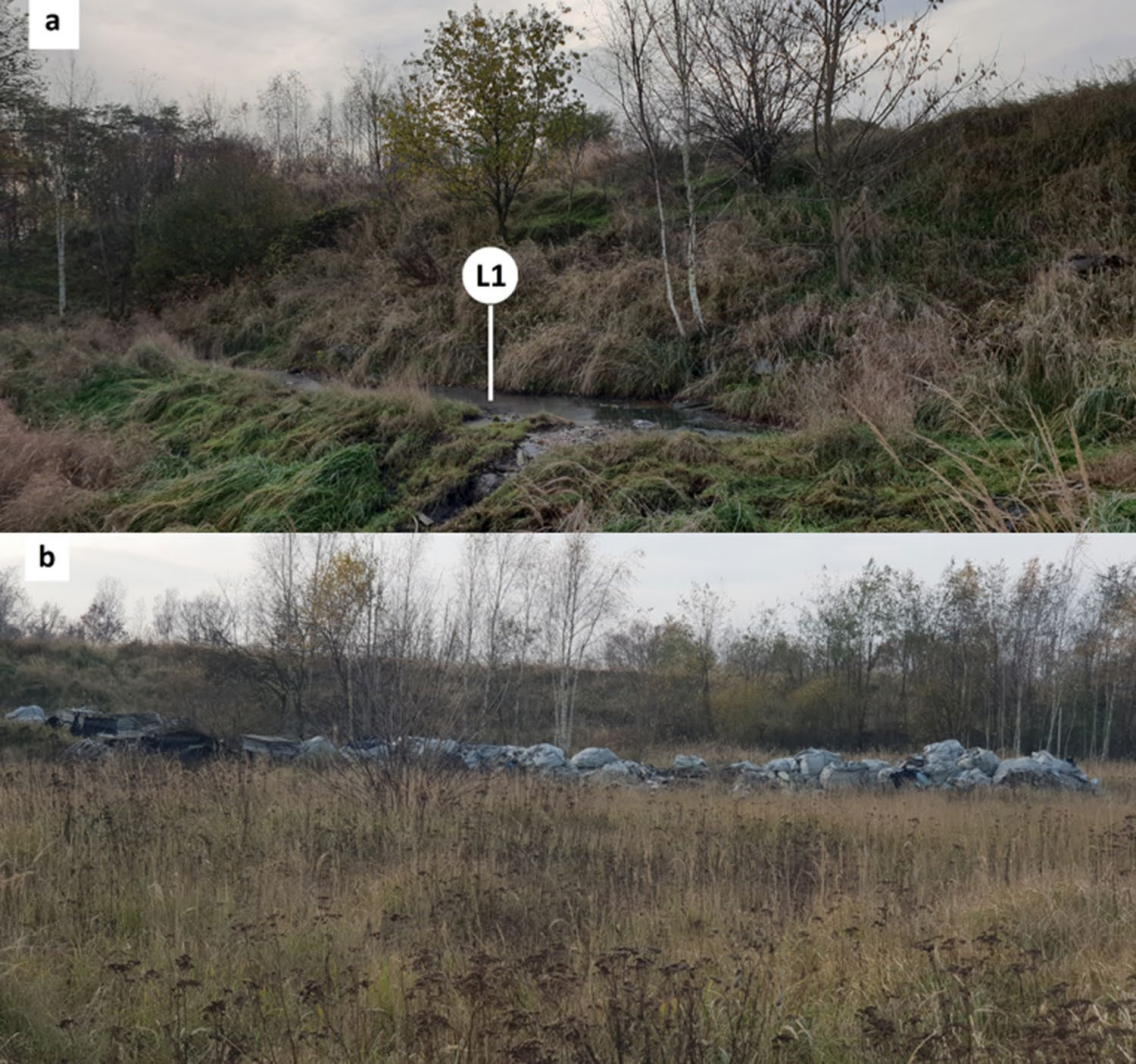
The closed landfill for hazardous waste of the former "Boruta" Dye Industry Plant with visible plant succession. The slope of the landfill near the leachate accumulation site L1 (**a**) and the surface of the landfill with asbestos bags (**b**).

The dye industry waste landfill is located within the town boundary of Zgierz (Fig. 1) near the former “Boruta” Dye Industry Plant (now a special economic zone with different industrial factories including dye houses). The landfill consists of one plot with an area of 0.81 ha and a cubature of 50,376 m^3^, limited by a dike^24^. It is adjacent to the closed energy ash and gypsum landfill. The total surface of the area is approximately 10 ha. The slopes of the landfill are 4—6.5 m high (Fig. 2a). The bottom of the storage basin and the slopes are sealed. The waterproofing was made of native soil in the form of sandy loams with a thickness of 5 to 10 m, on which a levelling layer of medium-grained sand was laid. Above it, a 2 mm thick geomembrane and a filtering layer were installed. At the bottom of the landfill basin, there are drainage pipes for the discharge of leachate from the landfill area^25–27^. Plant succession with grass predomination and growth of bushes and trees on the slopes of the landfill was observed on the surface of the landfill (Fig. 2a and b).

The dye industry landfill was explored in the years 1995 – 2015 mainly for the waste disposal of the former "Boruta" Dye Industry Plant in Zgierz. Postproduction waste of the former “Boruta” plant was stored in containers, barrels or bins and then covered with a layer of ash, sand or debris, as well as municipal waste with a pouring layer of ash, gypsum and bags with asbestos (Fig. 2b). The effluents from the landfill are introduced by the industrial sewage system of the former "Boruta" plant to the municipal and industrial wastewater plant in Zgierz. The plant receives (on average, per day) 10,000 m^3^ of urban wastewater and 1,500 m^3^ of industrial sludge, including liquid waste from azo dye production factories and landfill leachate. Cleaned wastewater is discharged to the Bzura River, which is a left tributary of the Vistula River that supplies water to the Baltic Sea (Fig. 1). The Baltic Sea is a relatively small (415,266 km^2^) semi-enclosed brackish water body, and the degree of contamination of the rivers flowing into it is of key importance for the biological balance of the sea^28^.

Due to the potential risk of water and soil contamination by landfill leachate, the chemical composition of the leachate, groundwater (with 9 piezometer applications), cleaned plant wastewater and water of the Bzura River are monitored by The Voivodeship Inspectorate of Environmental Protection in Łódź^19,20^. The results of the leachate study carried out in autumn 2020 are presented in Table 1.

**Table 1:** Parameters of leachate situated below the landfill which exceeded the permissible values included in the Regulation of ME^24^.

Parameter	Unit	Limit values	Samples and exceeded values
COD_Mang_^A^	mg L^-1^ O_2_	n.r	L1 462 ± 113
			L2 428 ± 114
BOD_5_^B^	mg L^-1^ O_2_	25	L1 400 ± 28.3
			L2 300 ± 13.6
Chlorides^A^	mg L^-1^ Cl	1,000	L1 2,138 ± 274
			L2 2,078 ± 78
Iron^A^	mg L^-1^ Fe	10	L1 23.8 ± 4.5
			L2 23.1 ± 4.4
Volatile phenols^A^	mg L^-1^	0.1	L1 0.45 ± 0.111
			L2 0.33 ± 0.082
*o*-tolidine^B^	mg L^-1^	n.r	L1 0.3 ± 0.01
			L2 0.43 ± 0.02
4,4’-oxydianiline^B^	mg L^-1^	n.r	L1 0.23 ± 0.01
			L2 0.03 ± 0.01
Electrolytic conductivity	µS cm^-1^	n.r	L1 10,150 ± 568
			L2 10,145 ± 568

n.r. – not regulated.

^A^The measurements were performed in the Main Research Laboratory in Łódź and presented in the Report no. 296/2020 prepared on behalf of the Voivodeship Inspectorate of Environmental Protection.

^B^The measurements were performed in the Department of Industrial Microbiology and Biotechnology, University of Lodz.

The analysis of the composition of leachates L1 and L2 shows that both the high content of TOC (total organic carbon) exceed the permissible values^29^ many times (799, 788 and 30 mg L^-1^ C, respectively). The increase in the TOC concentration occurred due to anthropogenic activity is one of the indicators showing the degree of water pollution. In the present study, the chemical oxygen demand (COD) and biochemical oxygen demand (BOD_5_) values were also high. Comparing the BOD_5_ and COD values determined in the same samples, it is possible to assess the susceptibility of textile wastewater to biodegradation as well as biodecolarization^30^. The BOD_5_ values of 400 and 300 mg L^-1^ O_2_ (for L1 and L2, respectively) suggest that both leachates can be easily biodegradable. However, the high content of chlorides and iron may limit the ability of fungal degradation^31^. Additionally, the amount of volatile phenols was 3–4 times higher than the limit value.

Attention should also be paid to the high values of electrolytic conductivity amounting to 10,150 and 10,145 μS cm^-1^. Piekutin^32^ found that the electrolytic conductivity in groundwater samples around municipal waste landfills ranged from 373.4 µS cm^-1^ to 998.8 µS cm^-1^, and it was concluded that mineral substances originating from mineral wastes deposited in landfills caused the results.

Our chromatographic study additionally revealed the following toxic aromatic amines: *o*-tolidine (0.3 and 0.43 mg L^-1^, respectively) and 3,3’- dioxyaniline (0.23 and 0.03 mg L^-1^, respectively) in both leachate samples L1 and L2. According to information obtained from the state and local control units (see Acknowledgements), aromatic amines have not been detected in the effluents released by the treatment plant into the Bzura River. However, the high amount of iron in leachate samples L1 and L2 (Table 1) indicates corrosion of the metal containers stored in the landfill. For this reason, there is a high probability of releasing much larger amounts of toxic amines and other harmful chemicals that will be introduced to the sewage treatment plant. Jasim et al.^33^ showed the presence of several aromatic amines (including benzidine and its derivatives, e.g., *o*-tolidine) in effluents collected from different stages of refinery industrial wastewater treatment plants and from the Tigris River around the station. The range of identified amines was from nondetected to almost 0.3 mg L^-1^. Benzidine, *o*‐toluidine, 3,3‐dimethylbenzidine, and 3,3‐dichlorobenzidine were detected by Mazzo et al.^34^ in river receiving wastewater from a textile industry, which was previously treated by the industry using activated sludge. This clearly shows that currently used wastewater treatment methods have a limited capacity for aromatic amine removal. Therefore, it is necessary to search for additional environmentally friendly methods and/or microorganisms that efficiently support the elimination of these dangerous pollutants.

### N. pironii growth in dye industry landfill leachate presence

**Figure 3:**
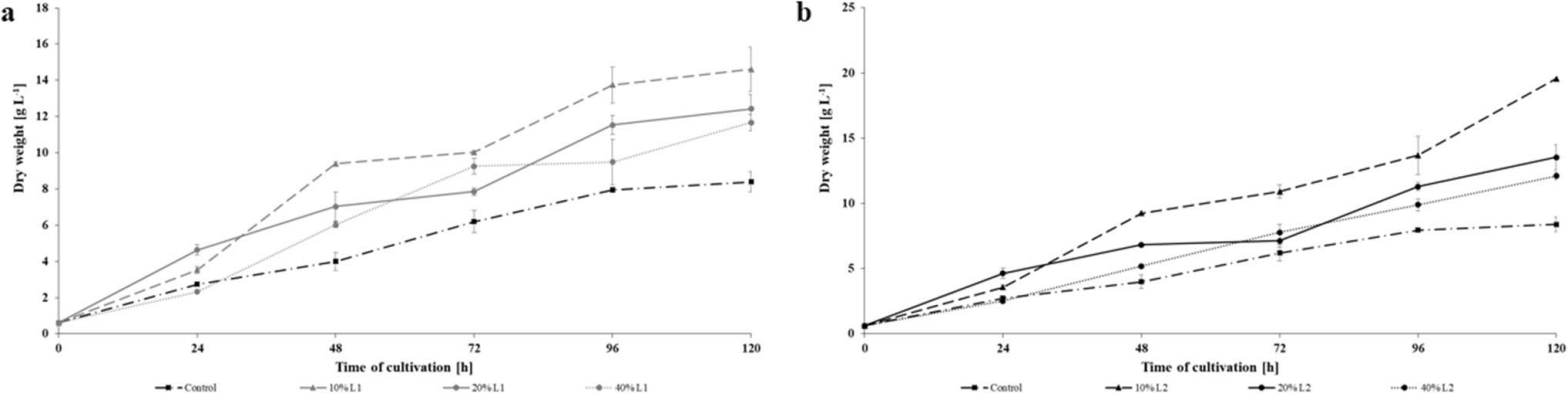
Dry weight of. *N. pironii* cultivated in Sabouraud medium containing 10; 20 or 40% of L1 (**a**) or L2 (**b**) landfill leachate or without leachate supplementation (control). Data points represent the means ± s.d., n = 3, P ≤ 0.05.

The results of *N. pironii* growth on Sabouraud medium supplemented with 10, 20 and 40% *v/v* leachate are presented in Fig. 3. The presence of leachate in the growth environment clearly favors the growth of fungal biomass. In the presence of 10% of the leachate, the biomass content in the culture after 120 h of incubation was almost twice as high for leachate L1 (14.6 g L^-1^) and more than twice as high for leachate L2 (19.6 g L^-1^). In the sets containing 20 and 40% of the leachate, a greater increase in the biomass of the fungus was also observed than in the control; however, it was lower than in the culture with the addition of 10% of the leachate. These data seem to indicate the presence of inorganic contaminants in both leachates slow down the biosynthesis processes at higher concentrations and be the cause of the lower growth at 20 and 40% of the leachate in the fungal cultures (Fig. 3).

### o-tolidine and azo dyes elimination

The leachate analysis (Table 1) also revealed the presence of aromatic amine *o*-tolidine and volatile phenols, which are used for the production of soluble azo dyes and insoluble pigments, particularly in the textile industries. From the mid-seventies of the last century until the restructuring of the dye production plants in Zgierz at the beginning of this century, the leading activity of the factory was combined with the benzidine department and synthesis of many kinds of azo dyes. Additionally, the new enterprises established on the site of the “Boruta” plants are involved in the production of this group of dyes^3,35^. The amount of *o*-tolidine in the effluents (Table 1) is relatively low (0.3 and 0.43 mg L^-1^ for L1 and L2, respectively), but the high iron content (23.8 and 23.1 mg L^-1^ Fe for L1 and L2, respectively) seems to indicate that this is corrosive to metal containers and that there may be a significant release of waste stored there, including aromatic amines, heavy metals and other components used for the synthesis of azo dyes. Therefore, the ability of *N. pironii* to grow and eliminate at higher concentrations of *o*-tolidine and following azo dyes was tested.

### o-tolidine biodegradation

Production of the dry mass of mycelium was assessed in cultures containing 0.5, 5, 10 or 50 mg L^-1^ *o*-tolidine, and the elimination of this compound from the growth medium was assessed using HPLC–MS/MS analysis.

**Figure 4:**
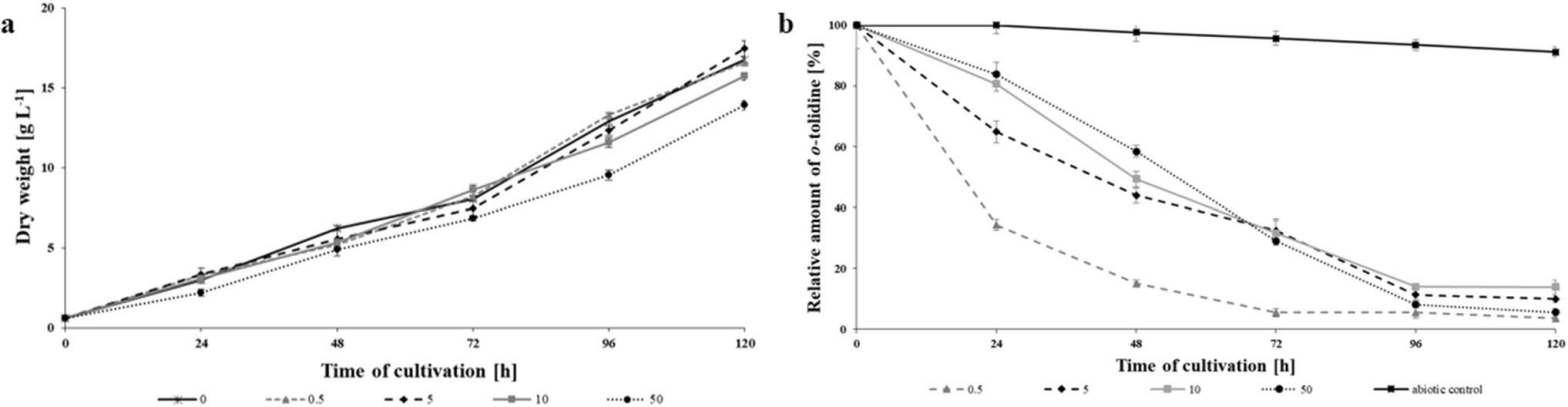
Dry weight of *N. pironii* (**a**) and *o*-tolidine residue (**b**) during the culture in Sabouraud medium with the addition of *o*-tolidine (0.5, 5, 10, 50 mg L^-1^ and abiotic control at a concentration of 50 mg L^-1^). Data points represent the means ± s.d., n = 3, P ≤ 0.05.

The microorganisms were able to grow in the presence of all tested *o*-tolidine concentrations (Fig. 4A). In the first 3 days of cultivation, the biomass determined in all test samples was similar and was in the range of 6–8 g L^-1^. After 72 h, growth inhibition of the fungus appeared in the cultures containing the highest concentration tested. In samples containing 50 mg L^-1^ *o*-tolidine taken after 96 h of cultivation, mycelium growth was limited by approx. 20%. At the same time, the time course of different concentrations of *o*-tolidine elimination was studied via HPLC–MS/MS analysis. It has been shown that microorganisms are able to eliminate *o*-tolidine from the growth medium at all tested concentrations (Fig. 4B). After 24 h of cultivation, almost 65% loss of the tested compound was found at the lowest of the tested concentrations. In cultures containing 5, 10 and 50 mg L^-1^ *o*-tolidine, the elimination after 24 h of culture was 35, 19 and 16%, respectively. A significant increase in the rate of *o*-tolidine degradation in these systems was observed after 96 h of cultivation. The xenobiotic content in the cultures initially containing 5, 10 and 50 mg L^-1^ decreased to 8–14% of the initial concentration and was 0.48, 1.34 and 4.11 mg L^-1^, respectively.

There are limited studies on aromatic amine biodegradation. Removal of these compounds was described mainly for aromatic amines from pesticides, drugs and dyes^36–39^. Various species of bacteria, e.g., *Bacillus* sp., *Pseudomonas* sp*., Proteus* sp., *Serratia* sp., *Enterobacter* sp. have been described as effective in aromatic amine biodegradation^40,41^. Previously, de Lima et al.^37^ reviewed papers describing “microbial bioremediation of aromatic amines” between 2015 and 2017 and found a lack of works employing fungal cells. Most of the work was on the biodegradation of primary or sulfonated amines.

**Figure 5:**
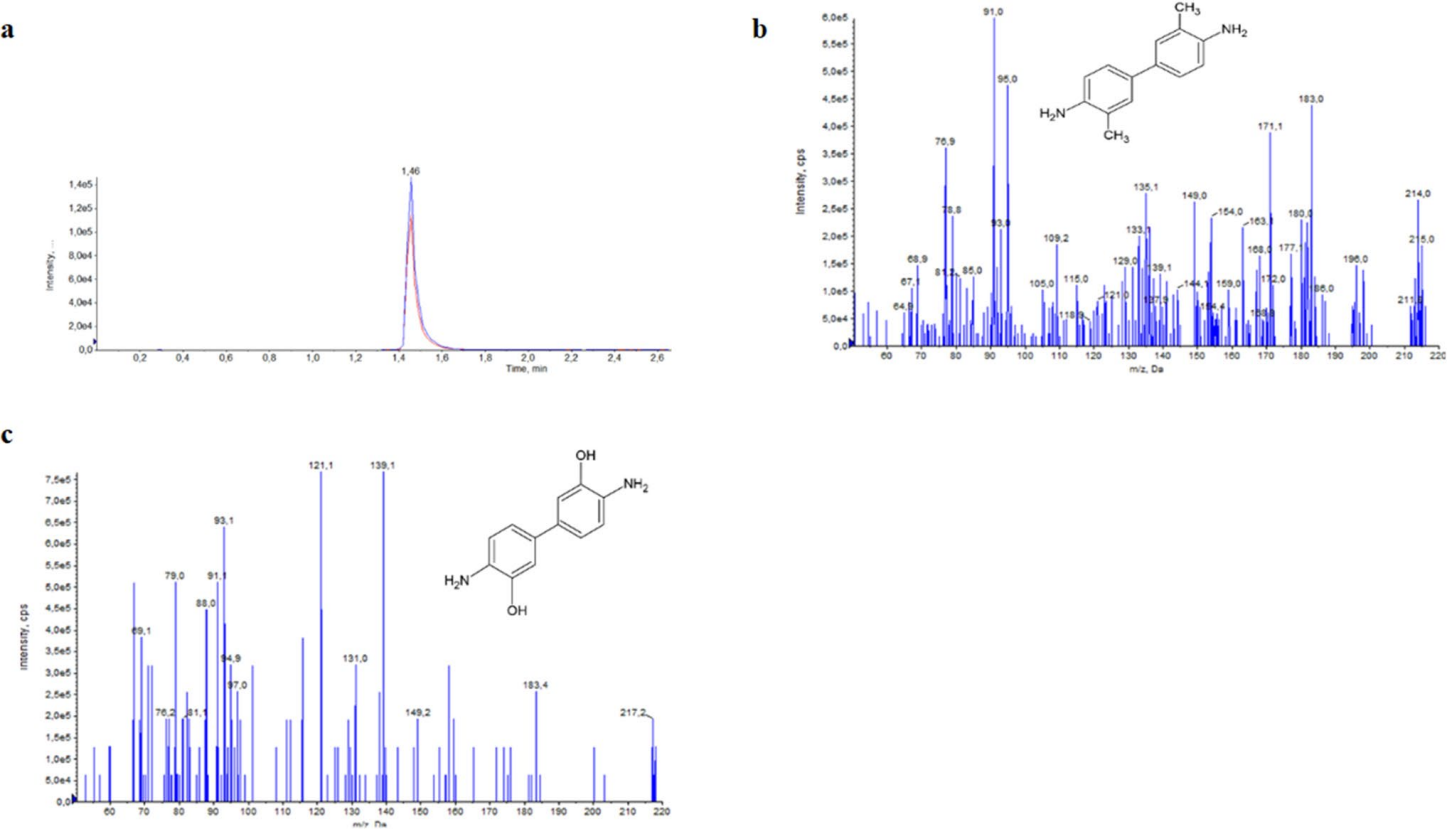
Chromatogram of *o*-tolidine (**a**), mass spectra of *o*-tolidine (**b**) and 3,3'-dihydroxybenzidine (C).

In the present study, *o*-tolidine was transformed mainly to less toxic 3,3’-dihydroxybenzidine (*m/z* 217) (Fig. 5). According to Brüschweiler and Merlot^15^, this compound gave negative results in the Ames screening test at concentrations ranging from 3–5,000 μg plate).

Additionally, in the cultures, acetyl *o*-toluidine (*m/z* 150.2) and *n*-hydroxy-*o*-toluidine (*m/z* 124) were identified. Mass spectra of the obtained metabolites were compared to the literature data. This suggests that *o*-tolidine may be hydroxylated (e.g., by extracellular laccase) and/or may be converted to toluidine and its derivatives. This is the first report on *o*-tolidine biotransformation by microscopic fungi. The obtained results may fill the gap in the research on fungal elimination of aromatic amines and may help in the development of efficient systems for the removal of these compounds from the environment.

### Azo dyes decolorization in the presence of heavy metals

The azo dye decolorization potential of the *N. pironii* fungus was assessed by examining its ability to degrade various dyes (Supplementary Table S1 online). The most efficient colour removal was observed for monoazo dye Acid Orange 7, followed by Reactive Red 120, a dye with a diazo bond. Just after 48 h of incubation, 79% of AO 7 and 75% of RR 120 were decolorized. Extending the cultivation time only slightly increased the level of decolorization, which indicates that dye elimination occurs mainly during the first 48 h of culture.

**Figure 6:**
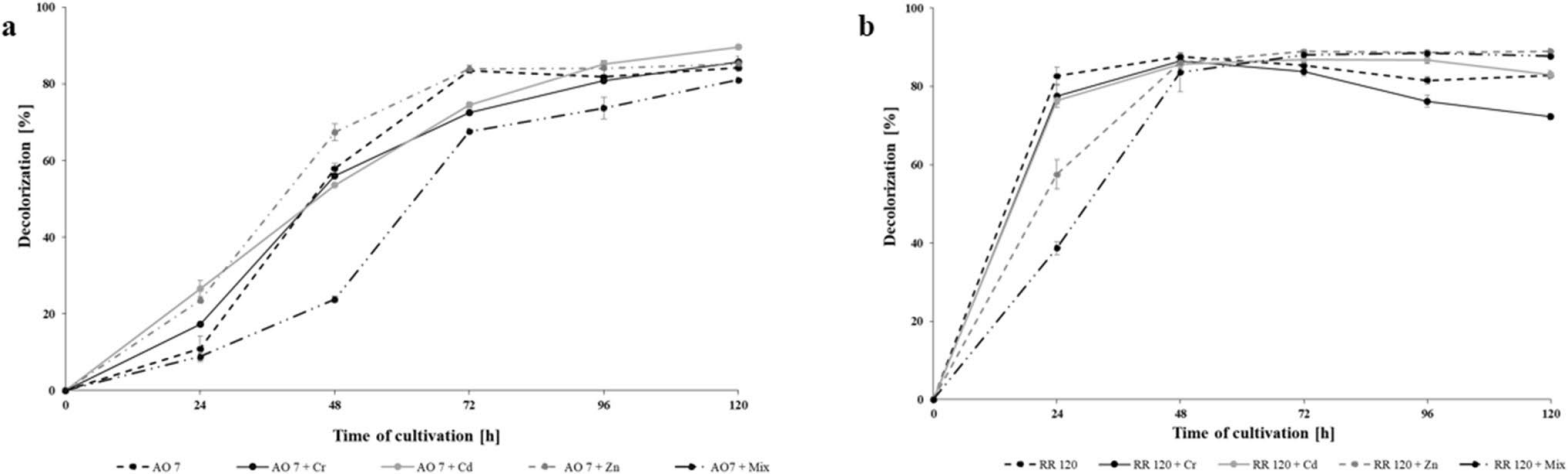
Decolorization of Acid Orange 7 (**a**) and Reactive Red 120 (**b**) at the concentration of 50 mg L^-1^ by the *N. pironii* culture in the presence of heavy metals (Cr(VI) 0.1 mM; Cd(II) 0.75 mM and Zn(II) 1.75 mM). Data points represent the means ± s.d., n = 3, P ≤ 0.05.

Considering that textile effluents are a mixture of pollutants containing dye residues, salts, heavy metals and many other chemicals used during dyeing, further study was carried out to examine the effect of selected metals on azo dye decolorization ability and *N. pironii* growth. The metals chosen were Cd(II), Cr(VI) and Zn(II), representing those commonly discharged in dye wastewaters^42^. The decolorization rate of RR 120 by *N. pironii* was affected mainly by Zn(II) added as a single metal (Fig. 6b). Decolorization noted in 24 h of cultivation in the presence of this metal was 24% lower than in the cultures conducted without metal addition. In turn, Cr(VI) and Cd(II) had only a very small or no effect on colour removal. When a metal mixture was used, decolorization was reduced by more than half. However, after 48 h of cultivation, no differences were observed in the tested systems. Initially, AO 7 decolorization was gently stimulated by the presence of the metals added individually (Fig. 6a). The presence of chromium, cadmium and zinc ions in the growth medium increased dye decolorization by 7%, 16% and 13%, respectively. However, these differences became less apparent in the next days of culturing. The addition of a mixture of all the tested metals significantly reduced the elimination of the dye by the fungus. This was particularly evident at 48 h of culture. The elimination of AO 7 in the presence of a metal mixture reached only 24%, while in the control system, the fungus removed 58% of the dye. Anwar et al.^43^ tested 38 bacterial strains for their potential to decolorize RR 120 in the presence of 25 mg L^−1^ Cr(VI). Only the strain identified as *Acinetobacter junii* showed the potential to simultaneously remove Cr(VI) and the selected azo dyes in the same medium. Similarly, the strain of *Pseudomonas* sp. showed good potential for decolorization of structurally diverse types of azo dyes in the presence of chromium, cadmium, zinc and lead ions. Furthermore, the bacterium reduced Cr(VI) by 41 to 95% along with concurrent decolorization of RR 120^44^. High dye removal (88–97%) was observed during growth, while the removal percentage for heavy metals ranged from 58 to 75%.

In real sewage from the textile industry, dyes are accompanied by other harmful compounds, e.g., heavy metals or amines used as dye precursors^45^. Therefore, the microorganisms used in dye biodegradation processes should also be resistant to the harmful effect of other substances present in sewage and should have the ability to perform biodegradation under such conditions^46^.

### Laccase and cytochrome P450 involvement in o-tolidine biotransformation

**Figure 7:**
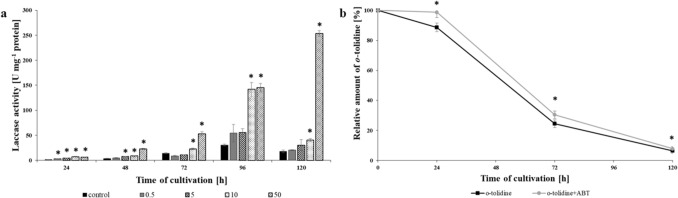
Laccase activity (U mg^-1^ of protein) during the *N. pironii* culture in Sabouraud medium with the addition of *o*-tolidine (0.5, 5, 10, 50 mg L^-1^) (**a**) and *o*-tolidine residue during the culture with *o*-tolidine (50 mg L^-1^) and 1-Aminobenzotrazole (ABT, 0.25 mM) (**b**). Data points represent the means ± s.d., n = 3, *indicates values that differ significantly from the control at P < 0.001.

*Involvement of laccase in o-tolidine biotransformation*Previously, *N. pironii* was described as an efficient laccase producer^23^. Thus, laccase activity was also estimated in the presence of *o*-tolidine at concentrations of 0.5, 5, 10 and 50 mg L^-1^. The results are presented on Fig. 7a. During the first two days of incubation with *N. pironii*, the laccase activity was low. However, after 72 h of incubation in cultures with the addition of 10 and 50 mg L^-1^ *o*-tolidine, the laccase activity was highest (22.8 and 52.7 U mg^-1^ protein) compared to the control (14 U mg^-1^ protein). Subsequently, laccase activity increased with increasing concentrations of *o*-tolidine. In the cultures with 10 and 50 mg L^-1^ *o*-tolidine, similar levels of laccase activity were achieved and were found to be 141.8 and 145.3 U mg^-1^ protein, respectively. The activity of the enzyme was the highest at 120 h in the presence of 50 mg L^-1^ *o*-tolidine and reached almost 253.4 U mg^-1^ protein.

In fungi, laccases are activated during the secondary metabolic phase of growth, which is caused by the depletion of nitrogen sources^47^. Although laccases are generally produced in low concentrations, supplementation of the growth medium with appropriate inducers such as metal ions, amino acids or aromatic compounds may increase their production and cause the production of new isoforms of enzymes^48,49^. Interestingly, an increased laccase activity in a *Trametes versicolor* culture was found to be supplemented with different compounds of industrial origin, such as nonylphenol or aniline^50^. The highest laccase activity in the presence of *o*-tolidine (10 and 50 mg L^-1^) obtained in this study may result from *o*-tolidine biotransformation and its metabolites, which affect enzyme production.

Laccase production is influenced by different factors (such as phenolic and nonphenolic inducer presence and carbon and nitrogen source depletion). Knowing that the biodegradation of *o*-tolidine involves laccase, it will be possible to control this process (increase its efficiency) by changing the environmental parameters and growth medium composition. In the future, the possibility of using pure *N. pironii* laccase for the elimination of *o*-tolidine and other toxic compounds derived from the textile industry will be tested.

### Cytochrome P450 monooxygenase involvement in o-tolidine biodegradation

Fungal cytochrome P450 monooxygenases, due to their versatile catalytic properties, are involved in many various and essential cellular processes. They catalyse the conversion of hydrophobic intermediates of primary and secondary metabolic pathways, detoxify natural and environmental pollutants and permit fungi to grow under different conditions^51,52^. Several investigations were conducted to determine cytochrome P450 involvement in the decolorization of dyes^53^ or elimination of xenobiotics. For example, our research team previously found involvement in the degradation of tributyltin (TBT) by *Cunninghamella elegans*^54^, 2,4-dichlorophenoxyacetic acid (2,4-D) by *Umbelopsis isabellina*^55^, 4-*n*-nonylphenol (4-*n-*NP) by *Metarhizium robertsii*^56^ or chloroxylenol (PCMX) by *C. elegans* IM1785/21GP^57^. The involvement of cytochrome P450 monooxygenases of the *N. pironii* strain in *o*-tolidine biodegradation was verified using the competitive inhibitor 1-aminobenzotriazole (ABT). As shown in Fig. 7b, after the first 24 h of incubation with ABT, the removal of *o*-tolidine was partially inhibited and its content reached 88.7%, while in cultures without inhibitor, it was 98.8%. Additionally, after 72 h of cultivation in the presence of ABT, *o*-tolidine residue was decreased to 24.5%, while in the control, it reached 30.4%. However, after 120 h, the elimination of *o*-tolidine was almost at the same level. The results indicate that cytochrome P450 monooxygenases might participate in the initial stage of *o*-tolidine degradation, but later, this process was not dependent on CYP450. In the white rot fungus *Phanerochaete chrysosporium* cytochrome P450 monooxygenases catalyse the oxidation of phenanthrene^58^ and pyrene^59^. The involvement of cytochrome P450 and laccase was studied by Nykiel-Szymańska et al.^60^. They proved that cytochrome P450 and laccase participate in the biotransformation of alachlor by *Trichoderma koningii*. The elimination of alachlor reached 90% after 72 h of incubation and even 80–60% in the presence of 1–5 mM copper.

## Conclusions

The present study revealed the ability of the ascomycete fungus *Nectriella pironii* originating from an urban postindustrial area to grow and retain metabolic activity in the presence of the dye industry waste. The fungus efficiently removed Acid orange 7 and Reactive Red 120 and hazardous aromatic amine (*o*-tolidine) used in azo dye production. This study documented for the first time *o*-tolidine biotransformation by microscopic fungi. Moreover, fungal laccase and cytochrome P450 involvement in this process has been confirmed. Additionally, the decolorization of azo dyes with the addition of heavy metals by *N. pironii* was studied. The high tolerance of the fungus to the tested hazardous compounds connected with their removal makes it an attractive tool in wastewater treatment plants applied for the elimination of dye industry waste. The presented results may also contribute to a precise understanding of the mechanisms controlling this process and promote the creation of a fungus-based system for the elimination of toxic pollutants from textile industry areas.

## Materials and methods

### Chemicals and landfill leachate

2,2′-Azino-bis(3-ethylbenzothiazoline-6-sulfonic acid) diammonium salt (ABTS) and 1-aminobeznotriazole (ABT) were obtained from Sigma-Aldrich (Germany). Stock solutions of ABT were prepared in ethanol at a concentration of 50 mM.

*o*-tolidine was purchased from Chempur (Poland). A stock solution of *o*-tolidine (10 mg mL^-1^) was prepared in ethanol. Liquid chromatography solvents were obtained from Avantor Performance Materials (Poland).

The azo dyes Reactive Orange 16 (CAS 12225–83-1), Acid Red 27 (CAS 915–67-3), Acid Orange 7 (CAS 633–96-5), Reactive Red 120 (CAS 61951–82-4), and Reactive Black 5 (CAS 17095–24-8) were purchased from Sigma-Aldrich (Germany). Stock solutions were prepared in water at a concentration of 50 mg mL^-1^.

Cd(NO_3_)_2_ × 4 H_2_O, K_2_Cr_2_O_7_, and ZnSO_4_ × 7 H_2_O were purchased from Sigma-Aldrich (Germany). Metal stock solutions were prepared by dissolving in deionized water at a concentration of 1 M.

Samples of landfill leachate (L1 and L2) collected from the landfill of the former “Boruta” Dye Industry Plant in Zgierz, Poland (Fig. 1) were kindly supported by the Voivodeship Inspectorate of Environmental Protection in Łódź, Poland.

### Strain and culture medium

The fungus *N. pironii* IM 6443 used in this study was previously isolated from a sample soil collected in the territory of the former “Boruta” Dye Industry Plant in Zgierz (a part of the Metropolitan Area of Łódź, Poland, Fig. 2) left to natural plant succession. The fungal strain was subcultured on ZT slants^61^ and maintained at 4 °C in the strain collection of the Department of Industrial Microbiology and Biotechnology, University of Łódź, Poland.

### Experimental setup and design

#### Fungal growth conditions

Fungal spores from 10-day-old cultures incubated on ZT slants were used for the preparation of pre-cultures in 25 mL of WHI medium^62^ using 100-mL Erlenmeyer flasks. The incubation was conducted at 28 °C on a rotary shaker (120 rpm). After 24 h, the pre-cultures were transferred to fresh WHI medium at a ratio of 1:4 and cultivated for 24 h^23^.

### Growth of N. pironii in medium with landfill leachates

Landfill leachates were centrifuged (6,000 × g for 10 min at 4 °C) and filtered to remove suspended solids before measurement. The cultures were prepared by inoculating Sabouraud dextrose broth liquid medium (BioMaxima, Poland) with 10, 20 or 40% *v/v* landfill leachate and 10% *v/v* inoculum. In addition, control flasks without added landfill leachate were included. All of the experiments were conducted in triplicate. Flasks were incubated on a rotary shaker (120 rpm) at 28 °C for 120 h.

### Analytical methods

#### Dry weight estimation of fungal biomass

The cultures in the flasks were filtered through pre-weighed Whatman filter paper No. 2 on a filtration set under slight vacuum, washed three times with distilled water and then dried at 105 °C in an oven until a constant weight^63^. The difference between the weight of dried filter paper with biomass and empty filter paper represented the fungal biomass. The results are expressed as mg mL^-1^, which corresponds to fungal biomass (mg) per volume of culture (mL).

#### *o*-tolidine extraction and analysis

After incubation, the fungal cultures were transferred into 50 mL Falcon tubes with 10 mL of acetonitrile and glass beads and homogenized with a ball mill (MM 400, Retsch) for 2 min. Next, QuEChERS salts (2 g MgSO_4_; 0.5 g NaCl; 0.5 g C_6_H_5_Na_3_O_7_ × 2 H_2_O; 0.25 g C_6_H_6_Na_2_O_7_ × 1.5 H_2_O) were added, and the tubes were vortexed for 2 min. Afterwards, the tubes were centrifuged for 10 min at 3,500 × g. After centrifugation, the upper layer was collected and transferred into an Eppendorf tube for chromatographic analysis.

### Determination of o-tolidine and its metabolites by HPLC–MS/MS

Measurement was performed using an Agilent 1200 HPLC (Santa Clara CA, USA) system and a 4500 QTrap mass spectrometer (Sciex, Framingham, MA, USA) with an ESI source. For reversed-phase chromatographic analysis, 10 μl of the diluted sample was injected onto a Kinetex C18 column (50 mm × 2.1 mm, particle size: 5 μm; Phenomenex, Torrance, CA, USA). The mobile phase consisted of 5 mM ammonium formate in water (A) and 5 mM ammonium formate in methanol (B). The solvent gradient was initiated at 20% B, increased to 80% B over 0.5 min, and maintained at 80% B for two additional minutes before returning to the initial solvent composition over 2 min. The column temperature was maintained at 40 °C, and the flow rate was 600 µl min^−1^.

The instrumental settings were as follows: spray voltage 5500 V, curtain gas (CUR) 25, nebulizer gas (GS1) 50, turbo gas (GS2) 50, ion source temperature of 500 °C, and positive polarization. Data analysis was performed with Analyst version 1.6.2 software (https://sciex.com/products/software/analyst-software; Sciex, Framingham, MA, USA). The quantitative analysis of *o*-tolidine was performed using multiple reaction monitoring (MRM). The monitored MRM pairs were *m/z* 213.3–196 and 213.3–181.

Tandem mass spectrometry for the identification of *o*-tolidine metabolites was performed using an enhanced MS scan (EMS) and precursor ion scanning (Prec). An information-dependent acquisition method, Prec (*m/z* 94) → EPI and EMS → EPI, was used to search ions corresponding to the protonated molecules [M + H]^+^.

### Landfill leachate analyses

The biochemical oxygen demand (BOD) of L1 and L2 was determined using the manometric respirometric BOD OxiTop (WTW, the Xylem Group, Germany). Samples were prepared according to European Norm^64^ and incubated in the dark for five days at a temperature of 20 °C.

The measurements of the landfill leachate quality were performed in the Main Research Laboratory in Łódź on behalf of the Voivodeship Inspectorate of Environmental Protection and evaluated by the following physical–chemical parameters: temperature^65^; pH^66^; conductivity^67^; COD_Mang_^68^; TOC^69^; nitrites^70^; nitrates, sulfates and chlorides^71^; antimony, arsenic, bar, beryl, boron, total chromium, zinc, aluminium, cadmium, cobalt, manganese, copper, molybdenum, nickel, lead, selenium, silver, thallium, titanium, vanadium and iron^72^; mercury^73^; free and bound cyanide^74^; petroleum hydrocarbons^75^; volatile phenols^76^.

### Assay of laccase activity and protein concentration

Laccase activity in the centrifuged supernatant was assayed by monitoring the oxidation of 10 mM ABTS (2,2′-azino-bis(3-ethylbenzothiazoline-6-sulfonic acid) diammonium salt) at 420 nm, as described previously^23,77^. One unit of laccase activity (U) was determined as the concentration of the enzyme required to oxidize 1 μM substrate per minute.

The protein concentration was determined using a bicinchoninic acid assay (BCA) test according to the Pierce BCA Protein Assay Kit protocol (Thermo Fisher Scientific).

### Cytochrome P450 inhibition study

1-Aminobenzotriazole (ABT)—cytochrome 450 inhibiting compound was introduced to the *N. pironii* cultures with the addition of *o*-tolidine (50 mg L^-1^) at the start of the cultivation to obtain a final concentration of 0.25 mM. Control flasks of *o*-tolidine (without inhibitor) were also prepared. All cultures were incubated on a rotary shaker (120 rpm) at 28 °C. The samples from 24, 72 and 120 h of cultivation were used in the experiment prepared in accordance with the method described in Section “*o*-tolidine extraction and analysis”.

### Characterization of the decolorizing potential of the N. pironii strain

Assays for the decolorization of dyes (RO 16, AR 27, RR 120, RB 5 and AO 7) at the initial concentration of 25 mg L^-1^ were performed in 18 mL of Sabouraud medium and inoculated with 10% inoculum. The cultures were prepared in 100-mL Erlenmeyer flasks and incubated on a rotary shaker (120 rpm) at 28 °C for 5 days. The abiotic controls of dyes and samples tested were carried out in triplicate. One milliliter of culture broth was sampled in an Eppendorf tube, centrifuged at 6,000 × g for 5 min and measured at the dye maximum absorbance wavelength (RO 16 λ_max_ = 492 nm; AR 27 λ_max_ = 529 nm, RR 120 λ_max_ = 516 nm, RB 5 λ_max_ = 598 nm, AO 7 λ_max_ = 467 nm) in a FLUOstar microplate reader (BMG Labtech, Germany) and Omega version 5.10 R2 software (https://www.bmglabtech.com/microplate-reader-software/)^78^. The percentage decolorization was calculated as Eq. (1):

1 $${\text{D }}\left[ \% \right] = \, \left[ {{1}00 \, \times \, \left( {{\text{A}}_{0} {-}{\text{ A}}_{{\text{t}}} /{\text{A}}_{0} } \right)} \right],$$

where A_0_ is the absorbance of the abiotic control, A_t_ is the absorbance of the culture supernatant, and D is the decolorization rate.

Additionally, the impact on decolorization rate of heavy metal compounds Cr(VI) 0.1 mM, Cd(II) 0.75 mM and Zn(II) 1.75 mM introduced to the *N. pironii* culture separately or as a mixture was measured as described previously.

### Spatial data analysis

Spatial data on the characteristics and field parameters of a landfill were obtained by the method of mapping degraded areas^79^ using GIS tools—Quantum GIS Bucuresti version 3.12 geoinformation software (https://qgis.org/pl/site/), OpenStreetMap and OSM Standard online GIS sources. Data on vegetation and landfill management were sourced from the field observation of the area in period 2019–2021 by using the methods of eco-urban documentation of green infrastructure^80^.

### Statistical analysis

Experiments were conducted in triplicate. The data values are presented as the mean ± standard deviation (SD). The statistical significance of differences between the mean values was compared using an analysis of variance (ANOVA) with Tukey’s post hoc test. The changes were considered significant at P < 0.001 in STATISTICA version 13.3 software (https://statistica.software.informer.com/13.3/; StatSoft).

## Supplementary Information

Supplementary Tables.
