# Supplementary material for: Environmental and molecular approach to dye industry waste degradation by the ascomycete fungus Nectriella pironii
Source: Sci Rep. 2021 Dec 13;11:23829. doi: 10.1038/s41598-021-03446-x (PMC8669018; doi:10.1038/s41598-021-03446-x)
Supplement: Supplementary file 1 — Supplementary Tables. [file 41598_2021_3446_MOESM1_ESM.docx]

**Environmental and molecular approach to dye industry waste degradation by the ascomycete fungus *Nectriella pironii***

Aleksandra Góralczyk-Bińkowska, Andrzej Długoński, Przemysław Bernat, Jerzy Długoński and Anna Jasińska

**Supplementary Table S1.** Decolorization of dyes (25 mg L^-1^) in the submerged cultures of *N. pironii.*

| **Time of cultivation [h]** | **Decolorization [%]** | | | | |
| --- | --- | --- | --- | --- | --- |
|  | **RR 120** | **AO 7** | **RB 5** | **AR 27** | **RO 16** |
| **48** | 79.31 ± 1.29 | 76.14 ± 0.93 | 26.44 ± 1.84 | 4.68 ± 1.60 | 0.31 ± 0.01 |
| **96** | 79.74 ± 4.29 | 78.28 ± 0.28 | 45.66 ± 2.52 | 5.21 ± 1.80 | 8.70 ± 0.22 |
| **120** | 80.80 ± 2.36 | 78.98 ± 0.67 | 56.20 ± 3.59 | 6.80 ± 1.90 | 11.29 ± 1.01 |
